# Subarachnoid Hemorrhage Caused by Supratentorial Cerebral Cavernous Malformation: A Case Report

**DOI:** 10.7759/cureus.51597

**Published:** 2024-01-03

**Authors:** Fatima Al Sada, Muhammad Mohsin Khan, Javeed Iqbal, Kazim Mohammed, Ali Ayyad

**Affiliations:** 1 Neurosurgery, Hamad Medical Corporation, Doha, QAT

**Keywords:** case report, ischemic stroke, non-aneurysmal subarachnoid hemorrhage, cavernoma, cavernous hemangioma

## Abstract

Cavernous malformations (CMs) are acquired vascular abnormalities of the central nervous system that are typically asymptomatic. Clinically symptomatic lesions may present with seizures, intracerebral hemorrhage, or focal neurological deficits. Very rarely, CMs have been described as the cause of subarachnoid hemorrhage.

We report a case of a previously healthy 58-year-old man who presented with acute onset of severe headache associated with vomiting. Head computed tomography (CT) scan showed subarachnoid hemorrhage with intraventricular extension. Subsequent CT angiography (CTA) and digital subtraction angiography (DSA) studies showed no evidence of vascular abnormalities. The patient was initially managed conservatively but later required neurosurgical and radiological interventions due to a complicated hospital course and worsening clinical condition. During surgery, an incidental mass was found in the temporal lobe, and subsequent histopathological examination confirmed the diagnosis of cavernoma, which was likely the underlying cause of the subarachnoid hemorrhage.

This report highlights the importance of considering CMs in the differential diagnoses of subarachnoid hemorrhage, especially in the absence of informative results from CTA and DSA studies. Timely detection and management of CMs may positively impact the clinical outcome, leading to reduced morbidity and mortality rates.

## Introduction

Cavernous malformations (CMs), also referred to as cavernomas, cavernous angiomas, or cavernous hemangiomas, are acquired vascular abnormalities of the central nervous system that may occur in the brain, spinal cord, or rarely, the dura [1]. They consist of clusters (caverns) of abnormally dilated, sinusoidal capillaries lined by endothelium [1]. CMs account for 5-15% of all vascular abnormalities of the central nervous system, with an estimated prevalence of 0.17-0.9% across populations [2]. They may be diagnosed incidentally or may present with seizures, intracerebral hemorrhage (ICH), or focal neurological deficits (FND) [3]. Unlike other forms of cerebral vascular abnormalities, CMs have rarely been described as the cause of subarachnoid hemorrhage (SAH), with less than 10 reported incidents over the past three decades. The majority of these cases involved infratentorial lesions, and only three cases were located in the supratentorial area. In this report, we present a case of a previously healthy man who developed non-traumatic, non-aneurysmal SAH due to a supratentorial cerebral cavernous malformation.

## Case presentation

**Figure 1:**
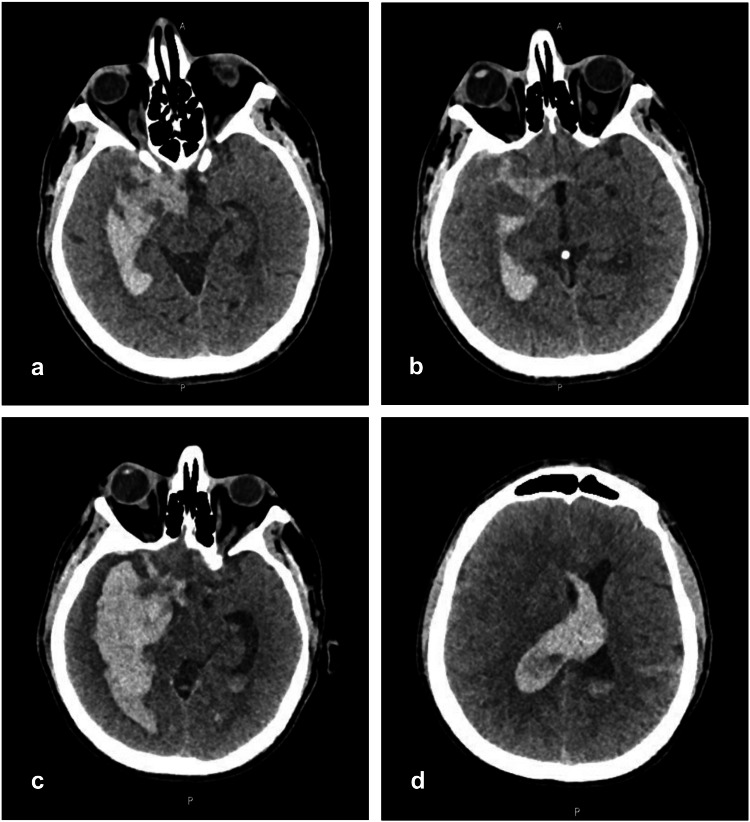
Noncontrast head computed tomography (CT) scan (a-b) Scan obtained on admission demonstrating subarachnoid hemorrhage in the suprasellar cistern with intraventricular extension; (c-d) Significant increase in the size of intraventricular hemorrhage with a midline shift of 10 mm

A 58-year-old man with no significant past medical or family history presented to the emergency department with a two-day history of an acute-onset severe holocephalic headache. On the day of presentation, the patient had three episodes of vomiting. Upon examination, vital signs were within normal limits, with a blood pressure of 130/70 mmHg. Glasgow Coma Scale (GCS) was 15/15 and neurological examination was unremarkable. A noncontrast head computed tomography (CT) scan was done, which showed a large intraventricular hemorrhage in the right lateral ventricle and suprasellar cistern, dilatation of ventricular and basal systems, and effacement of sulci suggestive of SAH (Figures 1a, 1b).

**Figure 2:**
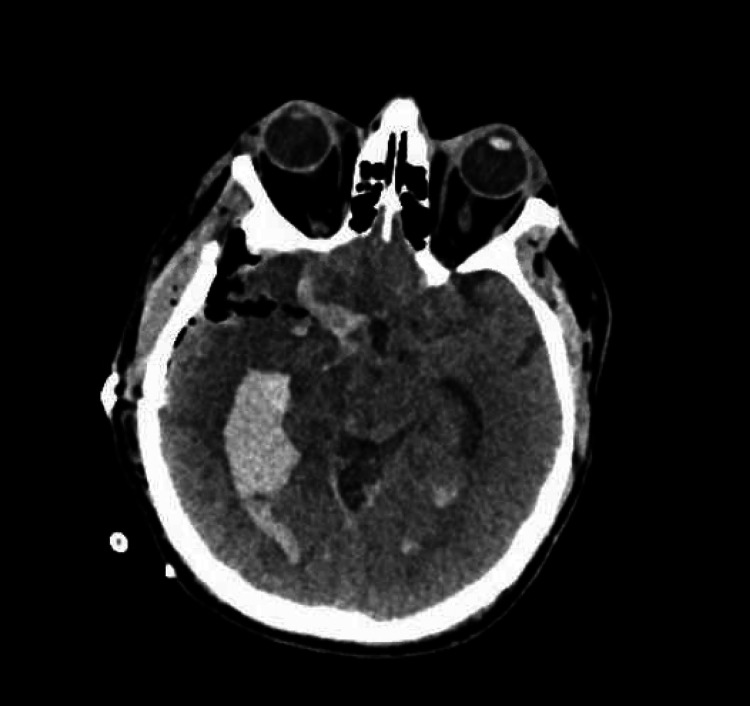
Postoperative noncontrast head computed tomography (CT) scan Postoperative CT scan of the surgical bed demonstrating hematoma evacuation and resection of the lesion.

Subsequently, CT angiography (CTA) and digital subtraction angiography (DSA) studies were done, both of which showed no evidence of cerebral vascular abnormalities. The patient was admitted to the surgical intensive care unit (SICU) under the care of the neurosurgery team for supportive management. However, early in his admission, the patient's condition deteriorated (E1V2M2), prompting an emergency CT scan that revealed further expansion of the intraventricular hemorrhage involving the right temporal lobe, dilation of the ventricular system, and a midline shift of 10 mm (Figures 1c, 1d). Consequently, the patient underwent a life-saving craniotomy for hematoma evacuation and external ventricular drain insertion (Figure 2).

**Figure 3:**
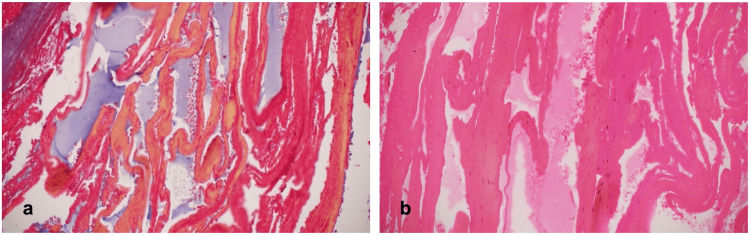
Histopathology of cavernous malformation Compact vessels of variable caliber and degree of wall collagenization with no significant intervening brain parenchyma and blood suggestive of cavernoma; (a) Masson’s trichrome stain x20; (b) H&E stain x20

During the operation, a firm, dark-red lesion was found on the roof of the anterior temporal hematoma. It was carefully dissected off the surrounding gliotic brain tissue and followed medially near the paraclinoid region. The lesion was near-completely resected and sent for histopathological examination, which confirmed the diagnosis of cavernoma (Figure 3).

Postoperatively, the patient remained in a critical condition with persistently low GCS (E4V1M5). On the sixth postoperative day, his condition further deteriorated (E1M5V1) and an emergency CT scan revealed newly formed right frontal cortical and subcortical hypo-densities, suggestive of acute infarct. Subsequently, CTA and CT perfusion studies were done, which demonstrated severe vasospasm of the right middle (M1, M2 segments) and anterior (A1 segment) cerebral arteries, and a small infarcted core with a larger penumbra, respectively. He was treated with intra-arterial nimodipine angioplasty, which resulted in significant improvement in the caliber of vessels and blood circulation. The patient remained in the SICU thereafter, with persistently poor neurological status and overall condition. As a result, the healthcare team held a meeting with the family members who opted for conservative management without further escalation in treatment and signed a Do Not Attempt Resuscitation (DNAR) form. On the twelfth postoperative day, brain death was confirmed and the patient was extubated.

## Discussion

In this report, we presented a case of a previously healthy middle-aged man who developed SAH complicated by cerebral vasospasm due to a supratentorial CM in an unusual clinical presentation. CMs are low-flow vascular lesions composed of clusters of dilated, leaky capillaries lined by endothelium that lack functional tight junctions. This results in abnormal vascular permeability that often leads to clinically asymptomatic microhemorrhages [2]. However, approximately 1 in 2000 patients may become symptomatic and present with seizures (20.8-36.0%), ICH (12-56.3%), or FND (6.5-15.2%) [3].

Spontaneous non-aneurysmal SAH (NASAH) is relatively uncommon and accounts for approximately 20-30% of all SAH cases [4,5]. Although the clinical outcome and prognosis of NASAH vary considerably due to the diversity of underlying causes and comorbidities, several studies showed that complications, including cerebral vasospasm, occurred less frequently in NASAH compared to aneurysmal SAH [6-8]. Around 10% of NASAH is caused by vascular malformations including, rarely, CMs [5]. In a prospective, population-based study, researchers investigated the radiological characteristics of intracranial hemorrhage caused by different cerebral vascular malformations [9]. All cases of CMs presented with ICH without extension to the ventricular or subarachnoid space, and none presented with isolated subarachnoid or intraventricular hemorrhage. In another study conducted on 820 patients presenting with non-traumatic SAH, 32 (3.9%) had atypical causes, out of which only one case was due to CM [8].

Over the past three decades, seven incidents of SAH due to CMs were reported in the literature, out of which only three were confirmed supratentorial lesions (Table 1).

**Table 1:** Summary of case reports ^* ^Multiple lesions were identified on imaging and/or postmortem examination, however, we mention the location of the lesion that caused a subarachnoid hemorrhage. ^⌜^Functional outcome at discharge only. HPE; histopathological examination

Author, Year	Age (years)	Sex	Location of Lesion	Presentation	Diagnosis	Management	Mortality	Functional Outcome ^⌜^
Yamamoto M., 1993 [10]	56	M	Cerebellum*	Headache, neck stiffness	MRI + HPE	Surgical	Alive	N/A
Takado, Y., 2009 [11]	62	F	Cerebellopontine angle	Headache	MRI + HPE	Surgical	Alive	No residual neurological deficits
Yaghi, S., 2011 [12]	70	M	Cerebellum*	Headache, diplopia	MRI only	Not reported	Alive	Not reported
Uneda, A., 2017 [13]	50	F	Cerebellum	Headache, vomiting, dizziness	MRI only	Conservative	Alive	No residual neurological deficits
Escott, E. J., 2001 [14]	40	F	Suprasellar*	Confusion, slurred speech	Postmortem HPE	N/A	Dead	N/A
Fernando, P. 2021 [15]	11	M	Occipital lobe	Loss of consciousness	Postmortem HPE	N/A	Dead	N/A
Thurman, C. 2022 [16]	33	F	Frontal lobe	Headache, photophobia, seizure	MRI only	Conservative	Alive	No residual neurological deficits
Present study	58	M	Temporal lobe	Headache, vomiting	HPE	Surgical	Dead	N/A

The first case of cerebral CM presenting with isolated SAH was described by Yamamoto et al. in 1993 [10]. They reported a case of a 56-year-old man with a family history of symptomatic CMs who presented with severe headaches. He was found to have a cerebellar lesion on MRI suggestive of cavernoma, which was confirmed postoperatively by histopathological examination. Three similar case reports described cerebellar CMs diagnosed by MRI in middle-aged and old patients presenting primarily with headaches [11-13]. In 2001, Escott et al. described a case of SAH caused by a suprasellar CM in a 40-year-old woman who presented with progressive confusion [14]. Recently, two case reports similar to ours described SAH caused by supratentorial CMs in different demographic groups. The first case reported by Fernando et al. described an 11-year-old boy who presented with loss of consciousness and was later found to have occipital lobe CM [15]. Thurman et al. described another case of a 33-year-old female with frontal lobe CM who presented with acute headache [16]. Among all reported cases, two were managed surgically, two opted for conservative management, two deteriorated before possible intervention, and one study did not report the clinical outcome.

There is great variability in the natural course and initial presentation of CMs across different cohorts, suggesting a possible interplay between genetic and environmental factors [3]. A recent prospective cohort study described several predictive factors of initial hemorrhagic presentation in CMs based on data from 202 patients followed over four years [17]. In this cohort, 37.1% presented with ICH. Results suggest that patients taking daily aspirin or any anti-thrombotic agent and non-steroidal anti-inflammatory medications were less likely to present with hemorrhage. Whereas brainstem location and estrogen use by women correlated with a higher likelihood of presenting with hemorrhage. Similarly, a meta-analysis of six studies with a total of 1620 patients showed that presentation with ICH or FND and brainstem location were associated with a higher risk of hemorrhage within five years of CM diagnosis [18].

The management of CMs remains a controversy due to the lack of randomized controlled trials, and current guidelines are largely based on observational data. In 2017, the scientific advisory board of the Angioma Alliance published consensus-based guidelines for the clinical management of CMs [19]. Generally, intracerebral and intraventricular hemorrhage caused by CMs should be managed according to the current guidelines for the management of spontaneous ICH due to other causes. Conservative management is recommended for asymptomatic lesions, but surgical resection of solitary lesions located in easily accessible non-eloquent areas may be considered. Neurosurgical intervention is recommended for symptomatic supratentorial lesions located in easily accessible areas, deep and brainstem lesions after a second symptomatic bleed, and lesions causing refractory epilepsy [19]. Radiosurgery and pharmacological agents are emerging alternatives that require further studies to synthesize high-quality, evidence-based recommendations.

In our case, the lesion was located in the supratentorial area, and the patient did not have potential risk factors for initial hemorrhagic presentation or poor prognosis. Our initial therapeutic approach involved supportive care and close observation for possible complications of SAH in accordance with evidence-based guidelines. Subsequent neurosurgical and radiological interventions were deemed necessary for life-saving purposes considering the complicated clinical course and poor outcome the patient had. Although MRI is the modality of choice for angiographically occult vascular abnormalities, including CMs, further use of radiological investigations was limited in our case due to the patient’s critical condition and clinical instability.

This report sheds light on an infrequent, yet important cause of subarachnoid hemorrhage; describing in detail the clinical trajectory and therapeutic considerations of the presented case, as well as similar cases reported in the literature. Additionally, the report draws attention to the potential diagnostic value of MRI in identifying CMs and other angiographically occult vascular malformations, which can present with non-traumatic, non-aneurysmal SAH. While effectively adding to the existing body of medical literature, results from a single case report are not sufficient to infer clinical associations and inform medical decisions. Further studies are essential to explore the prevalence of SAH due to CMs and comprehensively investigate clinical and patient-related factors in relation to disease morbidity and mortality.

## Conclusions

Despite the rare occurrence of subarachnoid hemorrhage secondary to cavernous malformations, it remains a possibility that carries serious and potentially fatal health consequences. Therefore, cavernous malformations and other angiographically occult vascular lesions should certainly be considered in the differential diagnoses of subarachnoid hemorrhage, especially in the absence of informative results from CTA and DSA studies. Early detection may guide the management plan and result in better clinical outcomes and decreased morbidity and mortality rates. We, therefore, suggest the use of MRI in the workup of such cases to rule out possible structural vascular etiologies.
